# The burden of visual impairment among Ethiopian adult population: Systematic review and meta-analysis

**DOI:** 10.1371/journal.pone.0288707

**Published:** 2023-07-20

**Authors:** Dilnessa Fentie, Yonatan Solomon, Tameru Menberu

**Affiliations:** 1 School of Medicine, College of Medicine and Health Sciences, Dire Dawa University, Dire Dawa, Ethiopia; 2 Department of Nursing, College of Medicine and Health Sciences, Dire Dawa University, Dire Dawa, Ethiopia; 3 Department of Medical Laboratory Science, College of Medicine and Health Sciences, Dire Dawa University, Dire Dawa, Ethiopia; Aravind Eye Hospital and Post Graduate Institute of Ophthalmology, INDIA

## Abstract

**Background:**

Visual impairment is a public health problem in both developing and developed countries, especially, in developing countries where most visually impaired communities are found. Knowledge of the pooled prevalence of visual impairment among adults is useful in planning, preventive programs and the provision of eye-care services for communities.

**Methods:**

Original observational studies published in English were included in this systematic review and meta-analysis. Eleven studies with a total sample size of 8,161 study participants were included. PubMed/Medline, HINARI, Google Scholar, Cochrane library, Web of Science, and African Journals Online databases were used to search for published articles. Data were extracted on a Microsoft Excel spreadsheet and analyzed using Revman 5.4.1 Software. Meta-analysis of further pooled prevalence estimates using the inverse variance heterogeneity model. The pooled estimate of visual impairment in the current review was estimated using a random-effects model. Forest plots were used to illustrate heterogeneity and to quantify the pooled prevalence of visual impairment. Publication bias was assessed using funnel plots. Visual impairment was defined as a presenting visual acuity of less than 6/18 in the betting-seen eye.

**Results:**

A total of 538 studies were identified from several Databases and digital libraries, of which eleven articles were selected for the final meta-analysis. Significant heterogeneity was observed across studies, suggesting a random-effects model to estimate the pooled prevalence of visual impairment. The prevalence of visual impairment in Ethiopia ranges from 10.3% in Addis Ababa central Ethiopia to 37.58% in the Northern Ethiopia. The pooled prevalence of visual impairment in the current review was 22% (95% CI: 17%–27%; I^2^ = 97%) using a random-effects model. There was also evidence of symmetry in the funnel plots.

**Conclusion:**

This systematic review and meta-analysis demonstrated a greater burden of visual impairment among Ethiopians in various study populations. Further, intervention strategies are required to reduce the burden of visual impairment and improve quality of life.

## 1. Introduction

Visual impairment (VI) is a functional restriction of the visual system that manifests as decreased visual acuity and the inability to see objects as clearly as a healthy person [1]. Moderate vision impairment, severe visual impairment, and blindness, as defined by WHO consultation, are defined as displaying visual acuity (VA) less than 6/18, 6/60, and 3/60, respectively [2, 3]. According to the World Health Organization (WHO), there are approximately 285 million visually impaired individuals worldwide, with 39 million being blind and 246 million having a low vision [4].

Globally in 2020, it was estimated that 237 million people had moderate to severe distance vision impairment. Eighty nine percent, they live in low- and middle-income nations, and 55% of them were women [5]. Furthermore, in the entire world, 216.6 million persons had moderate or severe vision impairment, with 118.9 million (or 55%) of those being female. In Africa, unaddressed vision impairment are estimated to be greater than 80% in western, eastern and central sub-Saharan Africa, while comparative rates in high-income regions of North America, Australasia, Western Europe, and of Asia-Pacific are reported to be lower than 10% [6].

The global burden of visual impairment is currently increasing, similar to an epidemic, with the greatest burden in middle- and low-income countries due to changes in lifestyle and the growing burden of non-communicable diseases. Decreased vision status is associated with increased morbidity and mortality, decreased quality of life, substantial economic productivity loss, and family stress [7].

The prevalence of blindness among all age Ethiopian population is found to be 1.18% (95% CI 0.650% to 1.706%) [8]. Furthermore, the prevalence of visual impairment in Ethiopia varies across sociodemographic and different study populations. Moreover, a study conducted in Addis Ababa, central Ethiopia showed that the prevalence of visual impairment was 10.3% whereas, another study conducted in northern Ethiopia showed that the prevalence of visual impairment was 37.58% [9, 10].

Although visual impairment is among the major public health challenges, studies addressing the magnitude of visual disorders in Ethiopian adults are insufficient and inconclusive. Therefore, this study aimed to estimate the pooled prevalence of visual impairments in Ethiopia.

## 2. Methods

### 2.1. Study design and search strategy

A systematic literature review and meta-analysis was conducted using published articles on the prevalence of visual impairment in Ethiopian adults. The search for articles was conducted between September 8, 2021 and February 27, 2022. This systematic literature review and meta-analysis were performed using the Preferred Reporting Items for Systematic Reviews and Meta-Analyses (PRISMA) [11] (see S1 File).

A Mendeley desktop was used to manage citations and to remove duplicate references. An extensive literature search was conducted to identify studies on the prevalence of visual impairment in Ethiopia. PubMed, Google Scholar, HINARI, Web of Science, Cochrane library and African journals were used to search for relevant papers in this review. Additional articles were found using a snowball approach. The search was done by using the following keywords: Visual impairment, Low vision, Ethiopian Adults, Visual disorder, Visual acuity status, alone and/or by using Boolean operators like “OR” or “AND”.

### 2.2. Selection criteria

Following the application of these criteria, studies were included if they (1) were published in the English language,(2) examined the prevalence of visual impairment, (3) were conducted in Ethiopia, (4) used a community or institutional-based study, and (5) age 18 years and above. We excluded (i) case reports, qualitative reports, comments, editorial letters, and reviews, and (ii) studies that did not report information relevant to the key outcome variable of visual impairment.

### 2.3. Operational definitions

**Visual impairment** was defined as presenting distance visual acuity worse than 6/18. It was further classified into moderate VI (presenting visual acuity (PVA) < 6/18–≤ 6/60), severe VI (PVA < 6/60–≤ 3/60), blindness (PVA < 3/60—NLP) [12, 13].

### 2.4. Data extraction and quality assessment

The selection technique was divided into two stages: First, titles and abstracts were screened against specified inclusion/exclusion criteria, and then the entire text of research reports selected as likely relevant in the initial screening was screened again. Two reviewers (D.F. and Y.S.) independently extracted the data using a Microsoft Excel spreadsheet and a standardized data extraction checklist. Mendeley desktop reference management was used to filter duplicate articles. The title, author name, year of publication, region (the area where the study was conducted), study design, outcome measurements, sample size, response rate, and number of participants with cases were all included in the data extraction checklist. Following the review by the two data extractors, any discrepancies were handled by a deep discussion for a possible consensus and mediated third reviewer (T.M.).

Two experts assessed the quality of the work using the Hoy 2012 technique, which included ten criteria for internal and external validity [14]. (1) population representation, (2) sampling frame, (3) methods of participant selection, (4) non-response bias, (5) data collection directly from subjects, (6) acceptability of case definition, (7) reliability and validity of the study tool, (8) mode of data collection, (9) length of prevalence period, and (10) numerator and denominator appropriateness were among the items of the Holy et al quality assessment technique. Unclear was regarded as having a high risk of bias. The overall risk of bias was then scored according to the number of high risk of bias per study: low (≤2), moderate (3–4), and high (≥5) (see S2 File).

### 2.5. Data processing and analysis

Data were extracted using a Microsoft Excel spreadsheet, and analysis was performed using Review Manager (RevMan) 5.4.1. The variance in the prevalence of visual impairment in each article was computed based on the binomial distribution formula by extracting the frequency sample size. The Cochran’s Q test and the inverse variance (I^2^) test statistic were used to examine heterogeneity among the studies. Cochran’s Q statistical heterogeneity test was considered statistically significant at p ≤ 0.01 and *I*^2^ statistics (at least 50% considered to be significant [15]. All outcome variables had a high degree of heterogeneity; hence, a random-effects model was used to estimate the pooled prevalence of visual impairment [16]. A funnel plot was used to examine publication bias [17]. A forest plot was used to describe the pooled prevalence with 95% confidence intervals.

## 3. Results

### 3.1. Study selection

**Fig 1:**
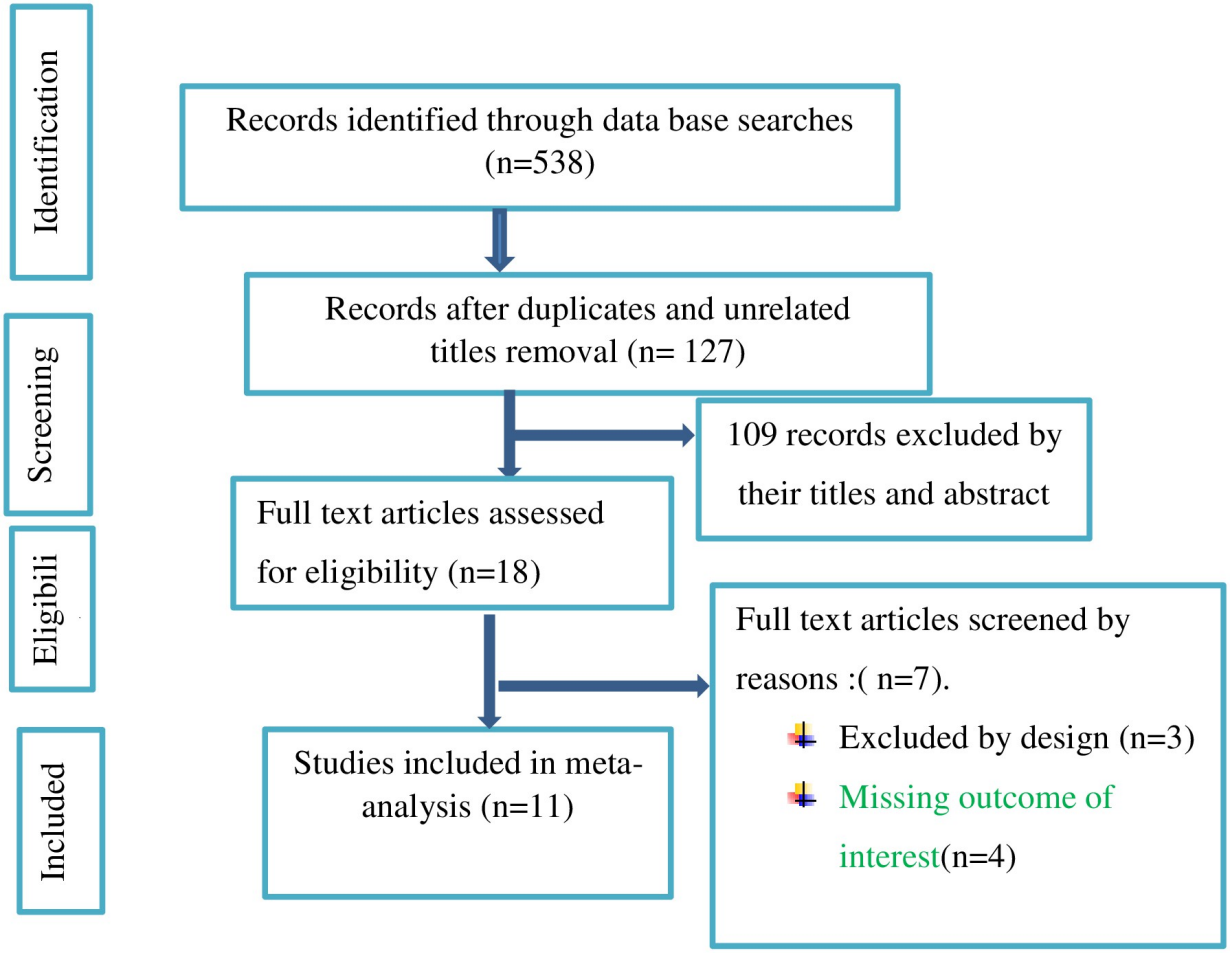
Flow diagram describing the steps of article inclusion to this systematic review.

We followed the PRISMA guideline to present the findings of this literature review and meta-analysis. After removing duplicate articles, 127 were screened, of which 109 were excluded after reading the titles and abstracts. The remaining 18 full text articles were assessed for their eligibility. Eleven studies met the eligibility criteria and were included in the final meta-analysis (Fig 1).

### 3.2. Study characteristics

Overall, we selected a total of 11 observational studies in this systematic review and meta- analysis. We included a total of 8,161 study participants to determine the overall prevalence of visual impairment in Ethiopian adult population. Out of the 11 studies, 9 of them were facility based [9, 10, 18–24], and the rest were community based studies [25, 26] (Table 1).

**Table 1:** Baseline characteristics of articles on the burden of visual impairment on the included studies (n = 11).

Author/year	Study population	Study design	Sample size (response rate)	Setting/facility or community	Prevalence of VI	Definition of VI
Haile W, 2015 [18]	Patients attending hospital	Crossectional	784(100%)	Hospital based	15.3%	VA<6/18
Cherinet et al, 2018 [9]	Patients attending hospital	Crossectional	881(97.4%)	Facility based	10.3%	VA<6/18
Diress et al, 2021 [19]	Pregnant women	Crossectional	417(98.6%)	Facility based	22.5%	VA<6/18
Abebe et al, 2021 [20]	Patients attending hospital	cross-sectional	312(96%)	Facility based	36.5%	less than 6/18
Seid et al, 2022 [10]	among type 2 diabetes patient	cross-sectional	322(97%)	Facility based	37.58%	VA<6/18,DR
Tiruneh et al, 2021 [21]	diabetic patients	cross-sectional	401 (100%)	Facility based	12.46%	VA<6/18,DR
Ketemaw Z, 2017 [22]	diabetic patients	cross-sectional	388(92%)	Facility based	29.38%	VA<6/18,DR
Getnet M et al, 2021 [23]	Medical and Health Science students	cross-sectional	654(100%)	Facility based	12.5%	VA<6/18
Temeselew et al, 2020 [24]	Patients Attending hospital	cross-sectional	715(100%)	Facility based	35.7%	VA<6/18
Melese M et al, 2003 [25]	General population	cross-sectional	2693 (90.8%)	Community based	12.1%	VA<6/18
Assegid Aga, 2001 [26]	General population	cross-sectional	571(100%)	Community based	18%	VA<6/18

VA = Visual acuity, DR = Diabetic retinopathy, VI = Visual impairment

### 3.3. Heterogeneity and publication bias

The risk of bias tool was used to assess the quality of each study using ten different items [14]. Our overall assessment of the 11 included studies found that eight had a low risk of bias (72.72%) [9, 10, 18–23], two of them had a moderate risk of bias (18.18%) [24, 26], and one had a high risk of bias (9%) [25].

**Fig 2:**
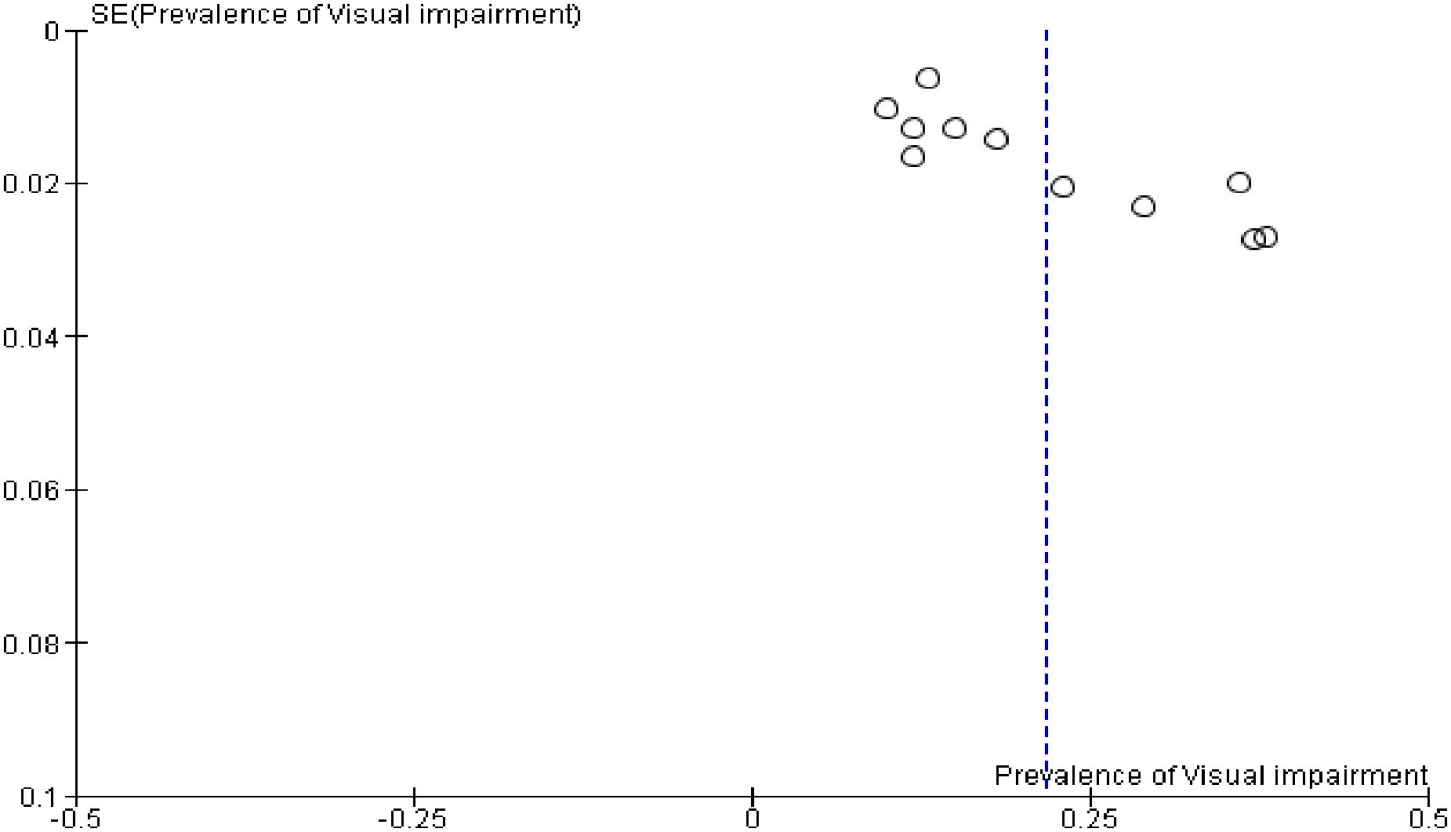
Funnel plot of 11 studies on the prevalence of visual impairment among the Ethiopian adult population.

The heterogeneity and publication bias of all eleven included studies were evaluated for visual impairment. As a result, the analysis showed considerable heterogeneity based on the Q test (p < 0.00001) and I^2^ statistics (I^2^ = 97%) which is indicative of the use of a random-effects model. There was no sign of publication bias or asymmetry in the funnel plots (Fig 2).

### 3.4. Prevalence of visual impairment in Ethiopia

**Fig 3:**
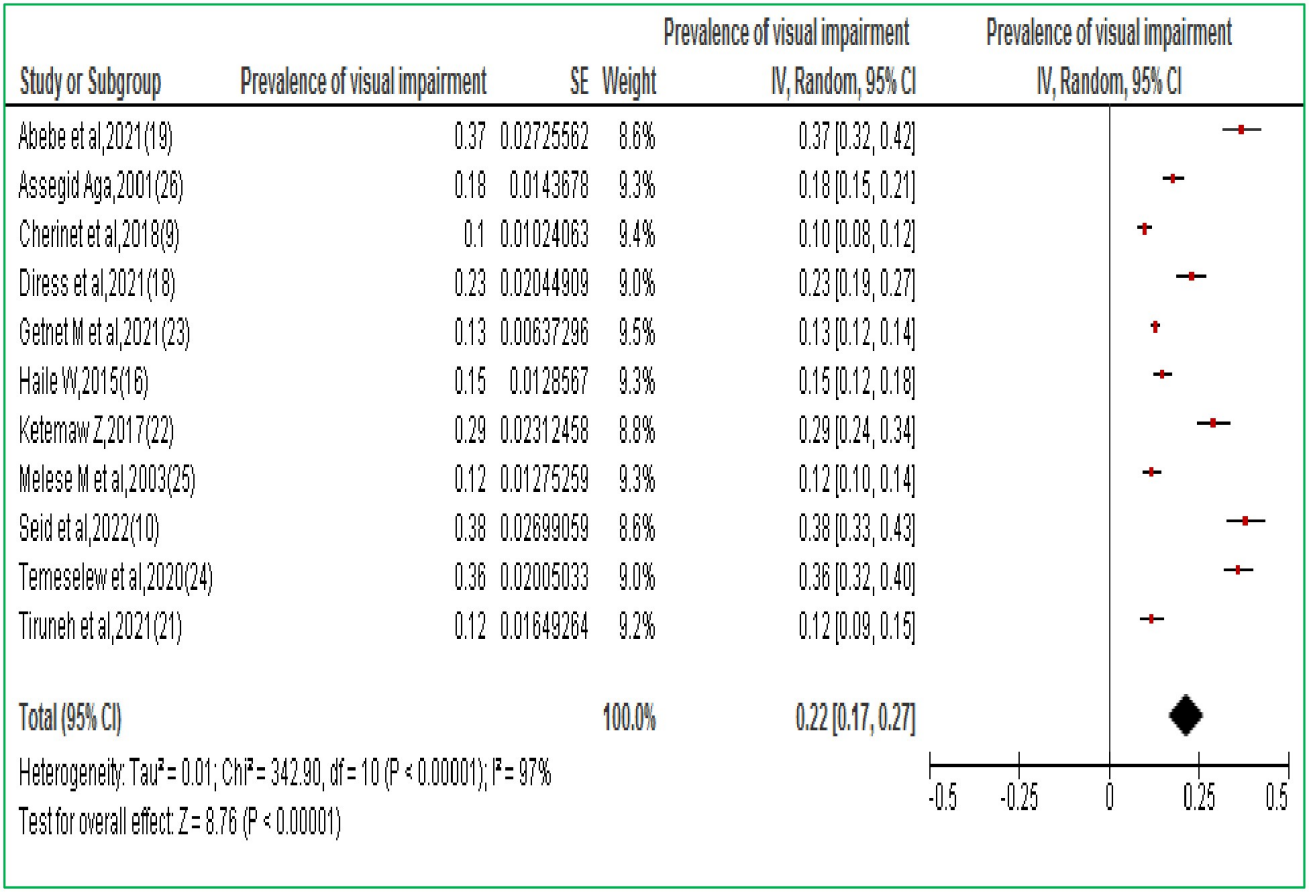
Forest plot of 11 studies on the prevalence of visual impairment among the Ethiopian adult population.

The burden of visual impairment in Ethiopia varies from 10.3% in Addis Ababa central Ethiopia [9] to 37.58% in the Amhara region northern Ethiopia [10]. According to the Der Simonian-Laird random-effects model, the overall prevalence of the meta-analysis of 11 studies, revealed that the pooled prevalence of visual impairment in Ethiopia was 22% (95% CI: 17–27%) (Fig 3).

## 4. Discussion

As per our knowledge, this is the only synthesis of observational studies that assessed the prevalence of visual impairment across different study population and study setting in Ethiopia.

Thirty-eight percent of adults with moderate to severe vision impairment are in their working years. This could make it difficult for individuals to find work, support themselves, and provide for their family. The increasing loss of independence is one of the most terrible outcomes of progressing visual impairment [27].

The current systematic review and meta-analysis provides evidence of an estimated pooled prevalence of visual impairment among various studies subjects of the Ethiopian population. The pooled prevalence of visual impairment was 22% (95% CI: 17–27%) which is in line with the study done in West Africa (17.1%) [28] and 14.1% in worldwide meta-analysis report [29]. On the other hand, our study is higher than meta-analysis study in England 3.6% [30], in United states 7.08% (95%CI: 6.32–7.89) [31], in Europeans 2.22% (95% CI: 1.34–3.10) [32], 5.57% (95% CI: 4.71–6.43%) in Iran [33], 10.9% (95% CI = 9.4%-12.6%) in China [34] and 4.2% in Nigeria (95% CI: 3.8%–4.6%) [35]. The discrepancy might be due to the time of the study, the cut of point to diagnosis visual impairment, types of study population, health-seeking behavior of the study participants, social-cultural differences, or access to health care services.

### 4.1. Limitations of the study

Even though the evaluation will be critical in presenting the overall burden of visual impairment in Ethiopia, it does have some limitations that should be recognized when it is used in future studies. Some of the papers included in this review had small sample sizes, which could have lowered the study’s power. Second, there are only studies from four Ethiopian regions limiting the study’s representatives. Furthermore, this study is not registered in PROSPERO, which may jeopardize the study’s transparency and trustworthiness. Even though our search method used systematic review searching, we could have inadvertently missed eligible studies due to restricted selection criteria.

## 5. Conclusions

In conclusion, regardless of the study subjects, gender, or classifications employed, our review concludes that a considerable proportion of the Ethiopian population has visual impairment. The pooled prevalence visual impairment is growing at an alarming rate and is high in Ethiopia. Therefore, policymakers, clinicians, and concerned stakeholders shall urge effective strategies in the control, prevention, and management of visual impairment.

## Supporting information

S1 File PRISMA checklist. (DOCX)

S2 File Risk of bias assessment of included studies using the Hoy 2012 tool. (XLSX)

## Abbreviations

AOR: adjusted odds ratio
ARMD: age-related macular degeneration
CI: Confidence Interval
COR: crude odds ratio
VA: visual acuity
VI: visual impairment
WHO: World Health Organization
